# Can local adaptation explain varying patterns of herbivory tolerance in a recently introduced woody plant in North America?

**DOI:** 10.1093/conphys/cox016

**Published:** 2017-03-28

**Authors:** Randall W. Long, Susan E. Bush, Kevin C. Grady, David S. Smith, Daniel L. Potts, Carla M. D'Antonio, Tom L. Dudley, Shannon D. Fehlberg, John F. Gaskin, Edward P. Glenn, Kevin R. Hultine

**Affiliations:** 1 Department of Ecology, Evolution and Marine Biology, University of California-Santa Barbara, Bldg 520, RM 4001, Fl 4L, Santa Barbara, CA 93106, USA; 2 School of Forestry, Northern Arizona University, S San Francisco St, Flagstaff, AZ 86011, USA; 3 Keck Science Department, Claremont McKenna, Pitzer and Scripps Colleges, 925 N. Mills Ave, Claremont, CA 91711, USA; 4 Biology Department, SUNY Buffalo State, 1300 Elmwood Ave, Buffalo, NY 14222, USA; 5 Marine Science Institute, University of California-Santa Barbara, Bldg 520, RM 4001, Fl 4L, Santa Barbara, CA 93106, USA; 6 Department of Research, Conservation and Collections, Desert Botanical Garden, 1201 N Galvin Pkwy, Phoenix, AZ 85008, USA; 7 USDA Agricultural Research Service, 1500 North Central Avenue, Sidney, MT 59270, USA; 8 Department of Soil, Water and Environmental Science, University of Arizona, 1428 E University Blvd, Tucson, AZ 85719, USA

**Keywords:** carbon allocation, climate change, *Diorhabda carinulata*, local adaptation, non-structural carbohydrates, *Tamarix*

## Abstract

Patterns of woody-plant mortality have been linked to global-scale environmental changes, such as extreme drought, heat stress, more frequent and intense fires, and episodic outbreaks of insects and pathogens. Although many studies have focussed on survival and mortality in response to specific physiological stresses, little attention has been paid to the role of genetic heritability of traits and local adaptation in influencing patterns of plant mortality, especially in non-native species. *Tamarix* spp. is a dominant, non-native riparian tree in western North America that is experiencing dieback in some areas of its range due to episodic herbivory by the recently introduced northern tamarisk leaf beetle (*Diorhabda carinulata*). We propose that genotype × environment interactions largely underpin current and future patterns of *Tamarix* mortality. We anticipate that (i) despite its recent introduction, and the potential for significant gene flow, *Tamarix* in western North America is generally adapted to local environmental conditions across its current range in part due to hybridization of two species; (ii) local adaptation to specific climate, soil and resource availability will yield predictable responses to episodic herbivory; and (iii) the ability to cope with a combination of episodic herbivory and increased aridity associated with climate change will be largely based on functional tradeoffs in resource allocation. This review focusses on the potential heritability of plant carbon allocation patterns in *Tamarix*, focussing on the relative contribution of acquired carbon to non-structural carbohydrate (NSC) pools versus other sinks as the basis for surviving episodic disturbance. Where high aridity and/or poor edaphic position lead to chronic stress, NSC pools may fall below a minimum threshold because of an imbalance between the supply of carbon and its demand by various sinks. Identifying patterns of local adaptation of traits related to resource allocation will improve forecasting of *Tamarix* population susceptibility to episodic herbivory.

## Teaser

Patterns of woody-plant mortality often reflect tradeoffs associated with resource allocation. Plants that allocate a high proportion of carbon acquired from photosynthesis to non-structural carbohydrate storage may be buffered from the synergistic effects of climate change and episodic disturbance.

## Introduction

Plant ecologists have recently paid considerable attention to woody-plant mortality because of continental-scale die-offs of woody plants across the globe (Allen *et al*., 2010). Rapid increases in mortality rates have been largely attributed to global environmental changes that have resulted in extreme droughts, heat waves, increased episodic insect and pathogen outbreaks and a measurable increase in forest fire frequency and intensity (Allen *et al*., 2010; van der Werf *et al*., 2010; Carnicer *et al*., 2011). Recent research has addressed the combined impacts of warming temperatures and water deficits on plant survival and there is now a wealth of data on the physiological mechanisms that underpin mortality surges in many regions (McDowell *et al*., 2008; Plaut *et al*., 2012; Sevanto *et al*., 2014). However, a clear genetic basis underlying reductions in plant fitness is still lacking for the expression of traits such as phenology, resource allocation or susceptibility to cavitation that are related to plant tolerance and resistance to environmental change. Understanding genetic variation in response to environmental change will improve predictions of future patterns of mortality across broad spatial scales.

A primary hurdle for addressing genetic versus environmental contributions to trait expression in plant mortality studies is that heritability is often difficult to measure in field settings. Common gardens provide excellent opportunities for testing hypotheses about traits that are favoured under specific environmental conditions. Numerous studies using common gardens of various species that incorporate genotypes from several source populations have yielded a broad range of information on genotype- and population-level patterns of net primary productivity (NPP), biomass allocation, water use efficiency, nutrient fertilization impacts on NPP and hydrologic processes (Zhang *et al*., 1993; Powers and Reynolds, 1999; Savolainen *et al*., 2007; Grady *et al*., 2011; Gray *et al*., 2016). Some of the strongest evidence that specific phenotypic traits are locally adapted to environmental conditions has emerged from common garden studies (Clausen *et al*., 1941; Savolainen *et al*., 2007; Grady *et al*., 2011). However, the relationship between local adaptation to a given stress and patterns of whole-plant mortality and canopy dieback under changing environmental conditions is largely unstudied (but see Yanchuk *et al*., 2008; Williams *et al*., 2014). This is particularly true for non-native plant taxa where few experimental common gardens have been established to address the potential for rapid selection on recently established populations (but see Alexander *et al*. 2012, Liao *et al*. 2016).

The genus *Tamarix* comprises a group of riparian woody species and their hybrids from Eurasia introduced to, and now distributed broadly across arid- and semi-arid regions of western North America. As with native woody plants in these regions, *Tamarix* spp. are experiencing moderate to extreme drought, and in addition exhibit dieback from episodic defoliation by a foliage-feeding beetle, *Diorhabda* spp. (Chrysomelidae), released for biological control of this genus (Bean *et al*. 2012). The northern tamarisk leaf beetle, *Diorhabda carinulata*, also native to Eurasia, was released more than a decade ago and has since affected thousands of hectares across the southwestern USA. The beetle produces two or more generations in a season and can develop high population densities that can completely defoliate *Tamarix* stands in <2 weeks. Repeated defoliation events eventually result in significant stand dieback and mortality (Pattison *et al*. 2011), but individual susceptibility to dieback can vary dramatically. Recent surveys across *Tamarix* populations have found that dieback can range from near 0% to >80%, after 2–5 years of repeated herbivory (Hultine *et al*., 2015a; Kennard *et al*., 2016). These disparate responses to repeated herbivory events invite many questions such as whether some *Tamarix* populations express phenotypic traits that make them more tolerant to episodic canopy disturbances than other populations. If so, two important follow-up questions (i) are there fundamental plant tradeoffs in carbon allocation patterns associated with herbivory tolerance? and (ii) is herbivory tolerance/sensitivity tied to adaptation to local environmental conditions? Although considerable research has been undertaken on *Tamarix* invasion into arid riparian ecosystems of North America, including identification of the hybrid nature of the genus in the western USA (Gaskin and Kazmer, 2009), information is lacking on the potential adaptive evolution of this highly successful non-native plant. Nevertheless, as beetle populations continue to disperse into broader geographic locations, clues are beginning to emerge on the extent to which *Tamarix* genotypes vary in their ability to cope with episodic foliage herbivory.

This paper synthesizes ongoing research on the patterns and mechanisms of *Tamarix* canopy dieback and mortality in response to intense episodic herbivory by *D. carinulata.* We focus on *Tamarix/Diorhabda* interactions as a model system to test hypotheses related to plant resource allocation, local adaptation and the impacts of multiple stressors on plant mortality and fitness. We present *Tamarix/Diorhabda* as a model system because of the intense episodic patterns of foliage herbivory by *Diorhabda*, coupled with the wide geographic distribution of *Tamarix* across broad environmental gradients and potential stressors. Together, these provide a system to investigate variation in traits associated with survival in response to defoliation events under a wide range of stressors. First, we provide a brief overview of the history of *Tamarix* in North America, followed by a review of recent research on the genetic diversity and evidence of local adaptation of *Tamarix* in its novel environment. We then propose potential tradeoffs associated with physiological traits related to carbon allocation. Here, we specifically address cases in which the expression of a given trait leads to resistance to one stress mechanism at the cost of reduced resistance to another stress mechanism, including stressors that are introduced or occur episodically, such as defoliation by *D. carinulata*. We pay special attention to the impacts that changes in mortality pressures can have on directional selection resulting in reduced genetic and phenotypic diversity, and potentially reduced tolerance of other stressors or competition. Finally, we summarize experiments that we believe are critical to merge studies of *Tamarix* mortality with those that focus on patterns of local adaptation, including the construction of common gardens and reciprocal transplant experiments. The specific hypotheses that are advanced here include: (i) despite its recent introduction, and the potential for significant gene flow, *Tamarix* in western North America is generally adapted to local environmental conditions across its current range in part due to hybridization of two species, (ii) local adaptation to specific climate, soil and resource availability conditions will yield predictable responses to episodic herbivory and (iii) the ability to cope with a combination of episodic herbivory and increased aridity associated with climate change will be largely based on functional tradeoffs in resource allocation that fall along a predictable trait spectrum.

## History and ecology of *Tamarix*

*Tamarix* has become one of the most successful non-native woody plants in the western USA, covering nearly 500 000 hectares (Friedman *et al*., 2005; Nagler *et al*., 2011), with a range that spans much of North America. Trees in this genus were introduced to the western states in the mid-19th century as ornamentals and for erosion control by governmental agencies due to their ability to thrive in xeric and saline environments (Horton, 1977). The genus was identified as a threat to native ecosystems in the 1930s (Robinson, 1965). Previously, the two most widely distributed species in North America, *T. ramosissima* and *T. chinensis*, were treated either as two separate taxa (Baum, 1978; Gaskin, 2003) or as a single aggregate species (Allred, 2002). These conflicting classifications are partly explained by results from molecular analyses that revealed that as much as 85% of *Tamarix* sampled from populations in the USA were a mosaic of hybrids between *T. ramosissima* and *T. chinensis* (Gaskin and Kazmer, 2009). While some hybridization of *Tamarix* species had been recognized in previous studies (Gaskin and Schaal, 2003; Gaskin and Shafroth, 2005), these two species and their related hybrids (hereafter referred to as *Tamarix*) are now recognized to dominate desert riparian habitats (Gaskin and Schaal, 2002; Sher, 2013). In addition to *T. ramosissima* and *T. chinensis* there are six other species of *Tamarix* in North America, and some of these hybridize with *T. ramosissima* and *T. chinensis* (i.e. *T. gallica, T. canariensis*, and *T. aphylla*), but these are less common species and even rarer as parents of hybrids (Gaskin and Schaal, 2003; Gaskin and Shafroth, 2005). *Tamarix* has had substantial impacts on hydrological function, the occurrence of fire and food web structure in riparian ecosystems in the southwestern USA and northern Mexico, in part due to the initial widespread planting of diverse *Tamarix* species (Friedman *et al*., 2005; Sher, 2013), as well as a suite of traits that allow *Tamarix* to be a rapid post-disturbance colonizer, a strong competitor and/or capable of tolerating considerable stress (Hultine and Dudley, 2013).

*Tamarix* has been targeted for large-scale removal projects in attempts to conserve water and maintain flows in arid regions based on assumptions that replacement of native taxa by *Tamarix* resulted in an overall increase of transpiration across riparian land areas (Shafroth *et al*., 2005). However, *Tamarix* removal from invaded systems has proved difficult, and control efforts using fire, mechanical removal at ground level, and herbicide treatments have mostly been proven to be ineffective or unsustainable (Gaskin, 2003). *Tamarix* resprouts from underground storage tissues following these eradication techniques, necessitating repeated treatments and sometimes soil reclamation to establish native species. The cost of removal alone is often prohibitive, around USD 1500–1700 ha^−1^ (Shafroth *et al*., 2005), and for successful eradication of established stands where revegetation and restoration efforts have been undertaken the cost can be upwards of USD 12 000 ha^−1^ (Zavaleta, 2000).

The USDA Agricultural Research Service began investigating the use of a biological control for *Tamarix* in the 1960s (Deloach *et al*., 2003). *Tamarix* seemed to be an ideal candidate for a biocontrol program, since there are no native congeners of *Tamarix* in North America (Gaskin, 2003). Three specialist insects from the *Tamarix* native range were approved by regulatory agencies for release, and beginning in 1998 controlled field trials began for the northern tamarisk leaf beetle (*D. carinulata*) to determine its suitability as a biocontrol agent (DeLoach *et al*., 2003). Open field releases followed successful cage trials in Colorado, Nevada and Utah that resulted in establishment and associated defoliation of *Tamarix* stands by *D. carinulata* (Dudley and Bean, 2012; Dudley *et al*., 2012); three other species of *Diorhabda* were subsequently released, primarily in Texas (Knutson *et al*., 2012). Multiple defoliation events typically occur over a single season, with up to three events at warmer sites (with longer growing season), and tree mortality has been documented after multiple years of defoliation (Bean *et al*., 2012). Some populations appear to be more tolerant to defoliation. For example, sites along the Humboldt River in Nevada tolerated three defoliation events per year for at least 3 years before any mortality was observed (Pattison *et al*. 2011). Likewise, sites along the Virgin River, a tributary of the Colorado River, have also exhibited low mortality rates after as many as seven defoliation events (Hultine *et al*., 2015a). By contrast, mortality of approximately half of individual plants has been documented at some sites along the Colorado River near Moab, Utah 3 years after the first observations of defoliation associated with *Diorhabda* feeding (Hultine *et al*., 2013). To date, the range of *D. carinulata* continues to expand southward, despite initial projections that physiological constraints would inhibit establishment south of the 38th parallel (Bean *et al*., 2012).

## Hybridization and the potential for local adaptation

Early investigators (Baker, 1965) suggested that invaders were successful because they had a ‘general purpose genotype’ that would perform well across a range of environmental conditions (Dlugosch and Parker, 2008). This hypothesis was grounded in the idea that founder effects reduce genetic variation in a species’ introduced range compared with its native range, and that populations of non-native species that become established would be composed of highly plastic individuals that could be successful across a range of environmental conditions (Baker, 1974). However, even early supporters of the ‘general purpose genotype’ hypothesis recognized that it could be offset by multiple introductions, or by plants with putative adaptive traits that are specialized to specific environments. It is believed that after initial colonization by a non-native species, local adaptation may play an important role in continued existence (Baker, 1974; Liao *et al*., 2016). There is building evidence that the hybridization of *Tamarix* may provide variation in traits that could promote local adaptation.

The hybridization of *T. ramosissima* with *T. chinensis* is widespread in North America (and southern Africa where the two species have also been introduced), but not in Eurasia in part because of the allopatric distributions in their native ranges (Gaskin and Schaal, 2002; Mayonde *et al*., 2016). Hybridization can also provide novel gene combinations that may promote adaptations to specific ecological problems in the new range that were not expressed by either parental lineage, as well as help overcome genetic bottlenecks that arise from founder effects (Anderson, 1953; Dlugosch and Parker, 2008). Furthermore, studies have shown that most naturalized *Tamarix* are more closely related to other wild naturalized *Tamarix* than they are to nearby ornamental populations, suggesting that most recent recruitment has occurred from naturalized individuals, despite potential gene flow between ornamental and naturalized populations (Gaskin and Kazmer, 2006). This suggests that naturalized genetic sources may be better adapted for successful invasion into these ecosystems than cultivated genetic lineages. Since *Tamarix* plants are fairly long lived (>70 years), and there has been extensive backcrossing, it is likely that hybridization occurred soon after introductions into western North America (Gaskin *et al*., 2012). Two pieces of evidence support the early hybridization hypothesis: (i) most individuals that have been sampled for molecular analysis from naturalized populations are the product of multiple generations of hybridization, as exhibited by varying levels of introgression (Gaskin and Kazmer, 2009); and (ii) studies of populations in southern Utah showed that the plants established there in the 1930s were *T. ramosissima* × *chinensis* hybrids (Gaskin *et al*., 2012). Nevertheless, information is generally lacking on whether breeding efforts by nurseries played a significant role in the observed patterns of hybridization in North America.

Molecular studies conducted across a latitudinal gradient from Texas to Montana showed that plants from lower latitudes were most representative of genetic material from the *T. chinensis* parental strain and that the higher latitude plants were most representative of *T. ramosissima* parents (Friedman *et al*., 2008; Gaskin and Kazmer, 2009). The climates in the extreme northern and southern ranges of the study reflect some of the differences between the climates of *T. ramosissima* and *T. chinensis* in their respective native ranges. Specifically, *T. ramosissima* occurs in the Eurasian interior where minimum temperatures are typically much lower than the thermal range of *T. chinensis* (Baum, 1978; Friedman *et al*., 2011). A common garden investigation of cold hardiness in *Tamarix* across this latitudinal gradient showed that plants from Montana were able to survive temperatures 21°C lower than the genotypes from Texas, and that there was a correlation between the latitude of origin and overwinter survival (Friedman *et al*., 2008). These data suggest that there has been local adaptation to climatic pressures (described in greater detail in the next section), and that hybridization of the two parental species may play a role in these adaptive traits as increased introgression towards *T. ramosissima* increased the cold hardiness of plants.

A recent genetic survey using microsatellites as described by Gaskin *et al*. (2006) and Friedman *et al*. (2008) was completed in 2016 from 18 Tamarix populations sampled across an elevation gradient in Arizona and southern Utah and showed that intra-population diversity was higher than inter-population diversity (Fehlberg, unpublished data). The amount of introgression towards *T. ramosissima* in these Arizona/southern Utah populations matched the levels of hybridization reported earlier from other locations in western North America (Gaskin and Schaal, 2002; Gaskin and Kazmer, 2009) and supports evidence that the majority of naturalized plants are hybrids of the two species. The novel genotypes created in such hybrid swarms could increase the capacity for emergence of a variety of genotype × environment combinations. Taken together, results from these genetic studies indicate that hybridization may explain the wide distribution and high abundance of *Tamarix* in the United States. Intermediate traits between the two parental species and novel gene combinations could provide the material for adaptation to local climatic and edaphic conditions or increase plastic expression of traits that allow for acclimatization to a range of sites by individuals. Variation in tolerance to cold, salinity and herbivory all indicate that hybridization could play an important role in the propensity of *Tamarix* to dominate riparian habitats.

## Evidence for local adaptation in *Tamarix* common garden studies

Local adaptation is driven by divergent natural selection on genotypes for traits that favour fitness in any given genotype × environment combination and should result in local populations that are more fit in their local habitat, defined by the suite of environmental factors, compared with populations from other habitats (Williams, 1966; Kawecki and Ebert, 2004). Gene flow, lack of genetic variation, extinction and environmental variability all have the potential to slow the emergence of local adaptation. Evidence has shown that gene flow can be maladaptive for populations, specifically away from the core of a species distribution range (Kirkpatrick and Barton, 1997), yet in small populations, increases in genetic variation resulting from gene flow can favour local adaptations (Moore and Hendry, 2009). Even in areas with high rates of gene flow, local adaptation may arise when there is strong selection due to high spatial heterogeneity in the environment (Macnair, 1991; Kawecki and Ebert, 2004) Multiple introductions may promote local adaptation by increasing the genetic diversity of a species in its introduced range, even potentially increasing diversity beyond that found in native ranges (Kolbe *et al*., 2004). This is especially true when introductions occur from across a large geographical distribution where plants may have low genetic diversity within a population but high diversity across populations (Dlugosch and Parker, 2008).

Native species have been shown to inherit traits linked to variation in cold hardiness and senescence periods, where selection over long periods of time have resulted in local populations that are more fit than conspecific individuals from other habitats in response to different photoperiods across latitudinal gradients and minimum winter temperatures in local climates (Howe *et al*., 1995). Similarly, introduced species can also express significant levels of local adaptation in their novel ranges (Rice *et al*., 1992; Dlugosch and Parker, 2008; Oduor *et al*., 2016). It has been shown that local adaptation can sometimes be as important as phenotypic plasticity in the capacity of some invasive plants to occupy a broad range of environments (Liao *et al*. 2016; Oduor *et al*. 2016). The bulk of literature available regarding local adaptation of non-native plants, however, has been based on annuals or herbaceous perennial species, and a paucity of work has been conducted with invasive perennial woody species.

Common garden studies involving *Tamarix* from populations exhibiting varying amounts of *T. ramosissima* introgression show that there is a range of traits that are expressed across a climate gradient (Friedman *et al*., 2008; Williams *et al*., 2014). A common garden study in Ft. Collins, Colorado compared characteristics of a native riparian tree species, *Populus deltoides* (Plains cottonwood), and *Tamarix* across a latitudinal gradient to investigate variations in cold hardiness and phenology (Friedman *et al*. 2008). As expected, the northern *P. deltoides* populations entered dormancy earlier and had higher rates of survival over the winter when compared with the southern populations. *Tamarix* populations showed similar results, with fewer individuals from the southern populations surviving through the winter (Friedman *et al*., 2008). Likewise, the date of leaf senescence was correlated with latitude for both *Tamarix* and *P. deltoides* genotypes in the common garden (Friedman *et al*., 2011). Studies have also shown that northern populations of *Tamarix* have a larger root to shoot ratio, indicating that northern populations allocate more biomass to belowground tissues than southern populations (Sexton *et al*., 2002; Williams *et al*., 2014). For populations experiencing higher rates of freeze-induced dieback, it may be adaptive to have larger pools of carbon in the form of belowground biomass for replacement growth of dead tissues when the growing season resumes. Similar trends of increased cold hardiness and early leaf senescence in *Tamarix* when compared with *P. deltoides* suggest that there may be rapid acclimatization of the introduced species to local environments.

**Figure 1:**
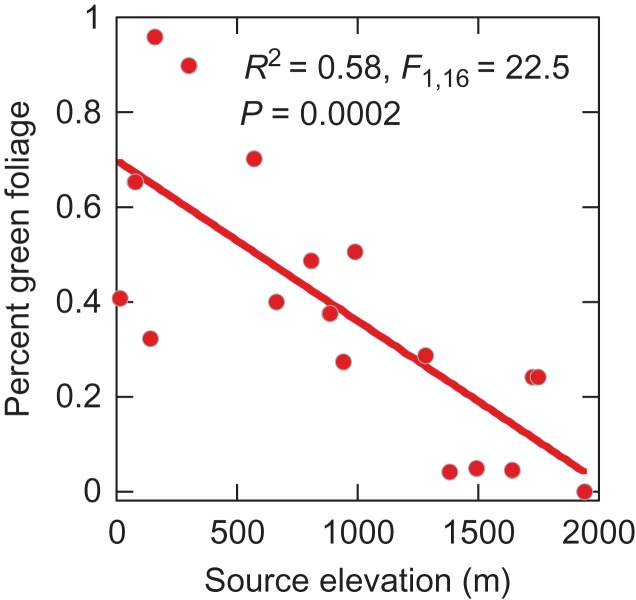
The correlation of percent green foliage versus population source elevation for *Tamarix* branches in January 2016 at a common garden in Yuma, AZ. Eighteen populations were represented at the common garden, collected across an elevation gradient from Arizona and Southern Utah. Populations originating from sites of lower elevation and correspondingly higher minimum winter temperature showed earlier leaf out relative to high elevation populations.

**Figure 2:**
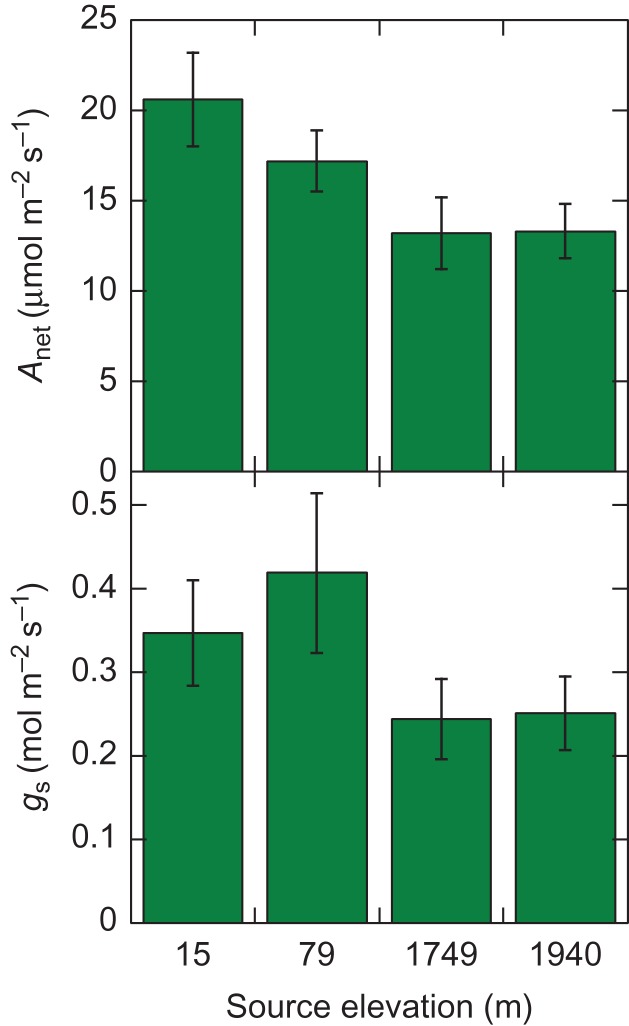
A comparison of mean ± SE net photosynthesis (*A*_net_, µmol CO_2_ m^−2^ s^−1^; upper panel) and mean ± SE stomatal conductance (*g*_s_, mol H_2_O m^−2^ s^−1^; lower panel) of four populations at a progeny study of *Tamarix* in Yuma, AZ. The common garden is located at an elevation of 56 m. Populations that were from nearby sites exhibited higher rates of photosynthesis and conductance compared with populations that were from higher elevations, showing some evidence for local adaptation based on differences in expressed traits in local and foreign populations.

Preliminary work from a *Tamarix* common garden study in Yuma, AZ also supports evidence of variation in phenology and foliar gas-exchange rates across populations. Phenology and leaf-level gas exchange patterns tend to correlate with minimum winter and maximum July/August temperatures of source populations. The common garden study conducted at the University of Arizona Mesa Farm (lat. 32.6151°N, long. −114.6365°W, elev. 60 m) involves 18 populations derived from an elevation gradient from 15 to 1940 m and a latitudinal range of 32.0° to 37.3°N. Beginning in January 2016, bimonthly phenological observations of floral characteristics were made on at least 36 individual plants from each population. Each individual was assigned a score for foliage greenness on a scale from 0 to 5, with a score of 0 indicating that the plants were still dormant, and a 5 indicating that the leaves were fully flushed out. Low elevation populations broke dormancy and were fully leafed out earlier than those that originated from mid- and high-elevation sites (Fig. 1). Likewise, leaf gas exchange measurements conducted in mid-June 2016 revealed that genotypes collected from low elevations sites with similar climate means as the common garden location in Yuma exhibited higher rates of midday net photosynthesis (*A*_net_) than genotypes collected from higher elevations with cooler climate means than in Yuma (upper panel of Fig. 2). The low-elevation populations also had higher rates of stomatal conductance (*g*_s_) when compared with high-elevation populations (lower panel of Fig. 2). Although the physiological mechanism that allows genotypes originating from warmer climate regions to maintain high levels of leaf-level photosynthetic rates is not known, the fact that phenology and physiology varied among provenances provides evidence that low-elevation genotypes may be adapted to the extreme heat that typifies low deserts of North America. Consequently, locally adapted Tamarix plants could become locally maladapted under projected future climate conditions.

## Fine-tuning carbon allocation to cope with multiple stressors

Plants face many challenges for avoiding mortality and maximizing fitness including competition, drought, disease and episodic disturbance from herbivory, fire and flooding. Compounding these challenges are changes in climate that are predicted to bring warmer temperatures, increased water deficits, alterations of fire regime and insect outbreaks, particularly in arid regions over the next century (Seager *et al*., 2007; IPCC, 2013). For trees, and other perennial plants, mortality is often not caused by a single stressor, but by multiple stressors interacting to reduce resource uptake and metabolic function below a minimum threshold for survival. For example, surges in forest mortality have been attributed to climate-induced stress coupled with insect outbreaks and fire (Kurz *et al*., 2008; Allen *et al*., 2010; Carnicer *et al*., 2011; Andregg *et al*., 2015). For many plants, the interaction between chronic drought and episodic disturbance leads to a cumulative reduction in resource supply relative to demand, particularly if already exposed to a long-term stressor such as poor edaphic conditions (Manion, 1991; Levanic *et al*., 2011). Therefore, persistence of individual plants and populations may depend on prior adaptation to stress, acclimation to edaphic and topographic location, as well as exposure to episodic disturbance events.

Regardless of edaphic location, disturbance and life history stage, plants must find ways to optimize carbon uptake and allocation by acquiring limited resources—mainly water, nutrients sunlight—under varying environmental conditions (Bloom *et al*., 1985). When resources are available above a minimum threshold, plants acquire carbon through photosynthesis and the products of photosynthesis (i.e. sugars) are used to maximize plant fitness—defined here as fecundity multiplied by life span. Maximizing fitness requires sugars to be allocated into a complex suite of carbon sinks, including tissue growth, reproduction, cellular respiration, defense chemistry (i.e. secondary metabolites and phenolics) and the storage of non-structural carbohydrates (NSCs) for subsequent utilization. Most fundamentally, osmotically active NSC pools maintain cell turgor and serve as a reservoir to buffer imbalances between carbon supply and demand (Chapin *et al*., 1990). Recently, particular attention has been given to the role NSC storage plays in overall plant function, fitness and capacity to withstand stress. These storage pools generally increase with plant size, which may serve as a major benefit since larger plants require larger buffers to cope with carbon imbalances in response to stress or episodic disturbance (Hoch *et al*., 2002; Sala and Hoch, 2009; Woodruff and Meinzer, 2011). Likewise, NSC pools are critical for vascular function (phloem and xylem) in response to varying environmental conditions. In fact, several studies have documented that NSC pools can play a primary role in maintaining plant water balance and long-distance water transport in the xylem by osmotically repairing xylem conduits following either drought-induced (Bucci *et al*., 2003; Nardini *et al*., 2011) or freeze-thaw-induced embolism (Woodruff and Meinzer, 2011). Given the importance of NSC pools for both carbon and water balance of long-lived woody plants, maintaining a minimum pool size may be a key trait for surviving resource limitations across various temporal scales.

**Figure 3:**
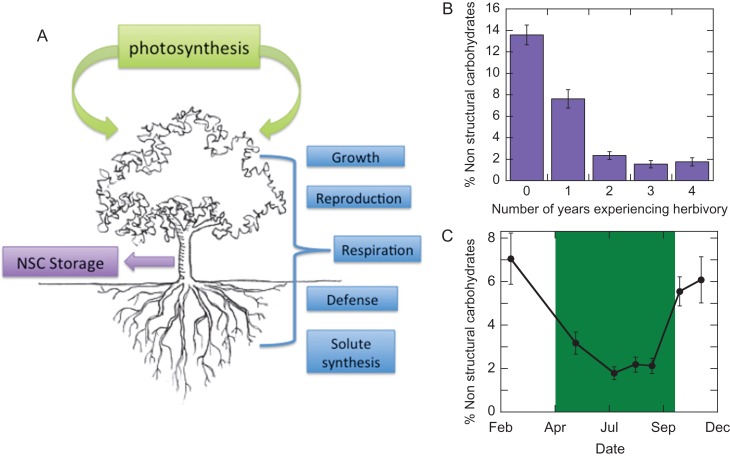
(**A**) A conceptualized carbon budget of woody plants showing the source of carbon coming from photosynthesis and the major carbon sinks. The conceptual diagram highlights NSC storage (purple box on the left) as a competing sink with all other sinks (shown on the right) including tissue growth, respiration (mitochondrial plus photo-respiration), reproduction, defense and solute synthesis. In *Tamarix* plants, the various carbon sinks that compete with NSC storage can be strong at different periods of the year given its typical environmental niche, trait expression and herbivory pressure. (**B**) (Redrawn from Hudgeons *et al*., 2007): Mean percentage of NSC storage measured in the root crown of mature *Tamarix* trees in northern Nevada. The trees had been exposed to a range of 0–4 years of episodic herbivory by the northern *Tamarix* leaf beetle (*Diorhabda carinulata*). Error bars represent the standard error of the means. (**C**) (Redrawn from Hultine *et al*., 2015b): The seasonal pattern of NSC concentrations in the twigs of mature *Tamarix* trees occurring in southeastern Utah (*n* = 20 trees). The patterns show a reduction in NSC storage during the growing season, revealing competing sinks between growth and storage as shown in the schematic on the left of Figure 3.

Traditionally, NSC storage was viewed as a consequence of weakening carbon sinks from reduced growth and respiration near the conclusion of the growing season when there was a surplus of carbon being acquired (Chapin *et al*., 1990). Recent evidence, however, suggests that NSC storage may be highly regulated and is often a competing sink for recently acquired carbon throughout the growing season (Fig. 3) (Hoch *et al*., 2003; Sala *et al*., 2012). Active regulation of carbon allocation may be coordinated by a complex genetic linkage among traits related to photosynthesis, growth, storage and other carbon movement (Smith and Stitt, 2007). Therefore, carbon allocation strategies could be highly variable within and among plant populations, depending on selective pressures from competition, resource limitation and disturbance. For example, if total carbon pools are equal between two plants (i.e. similar rates of total carbon assimilation *via* photosynthesis), then one plant demonstrating greater tissue synthesis and subsequent growth will do so at the expense of having less photosynthate to allocate to other sinks including NSC storage (Fig. 3). In locations where competition for sunlight is high and episodic disturbance is low (e.g. tropical rainforests), natural selection should favour individuals who maximize growth at the expense of NSC storage. Conversely, where competition is low, but disturbance or stress is high, or resource availability fluctuates, natural selection should favour individuals who allocate more of their photosynthate to NSC storage. However, plants that grow in riparian ecosystems, including *Tamarix*, are often exposed to competition, stress and episodic disturbance, requiring a high level of functional diversity in physiological traits. Therefore, riparian plant species will likely express a range of carbon allocation strategies, particularly in locations where there is strong gene flow among populations.

The biocontrol agent, *D. carinulata* and other recently released *Diorhabda* species are exerting intense herbivory pressure on *Tamarix* throughout the southwestern US, resulting in a potentially significant shift in what could be optimal plant carbon allocation. *Tamarix* has evolved under intense pressure from herbivory: in its home range, *Tamarix* is attacked by >320 species of insects and mites from 88 genera (Kovalev, 1995). This plant–insect co-evolution has likely contributed to a diverse set of strategies to cope with damaging insects. Among these strategies could be one in which *Tamarix* plants reserve relatively large pools of NSC with which to replace damaged tissues following herbivory (Hultine *et al*., 2015b), although these pools can be rapidly depleted following recurrent episodic herbivory (Hudgeons *et al*., 2007; Fig. 3A). As mentioned above, replenishing NSC pools comes at the cost of reduced allocation to other sinks (Fig. 3B), especially if canopy-scale photosynthetic capacity is reduced as a consequence of foliage herbivory (Strauss and Agrawal, 1999; Pattison *et al*., 2011). Herbivore pressure by *Diorhabda* is, nonetheless, a new phenomenon in North America where previously there had been little selection for herbivory tolerance in *Tamarix* plants. This shift in selective pressures could result in a potentially significant change in optimal plant carbon allocation. However, desert riparian settings require physiological traits to best cope with a combination of competition, temperature extremes, salinity and stochastic recruitment opportunities (Table 1). These selective pressures will, at least in part, favour allocation to growth, respiration (growth and maintenance respiration), reproduction and solute synthesis (Box 1), all at the potential expense of reduced allocation to NSC storage. Thus, plants that exhibit allocation to traits associated with success in the most taxing desert riparian settings (e.g. high salinity with extreme temperatures) may be maladapted to deal with herbivory-caused reduced NSC storage compared with individuals growing in low stress sites.

**Table 1:** Common environmental conditions *Tamarix* plants face during their life history and the carbon allocation strategies required to maximize fitness and survival under specific conditions

Environmental condition	Carbon cost
Insect/pathogen infestation	Resistance from defensive chemistry/secondary metabolite production
High soil/groundwater salinity	Solute synthesis to osmotically exude salts from leaves
Increasing depth to groundwater	Tissue construction for rapid root growth
Intra–inter-specific competition for sunlight	Tissue construction for rapid canopy growth rates
High growing season temperature	High mitochondrial respiration rates
Stochastic recruitment opportunities	Continuous flower and seed production to cope with unpredictable soil moisture conditions
Potential growing season freezing events	High NSC concentrations for tissue growth following frost-induced dieback
High fire frequency	High NSC concentrations for resprouting following episodic fire
High episodic foliage herbivory	High NSC concentrations to construct new foliage following herbivory events

Box 1. **Mechanisms and costs of salinity tolerance in *Tamarix***

Halophytes, such as *Tamarix* spp., are defined as plants that have a high tolerance to salt. Generally speaking, there are three distinct ways that plants tolerate high salt concentrations (Munns and Tester, 2008): (i) tolerance to osmotic stress; (ii) salt exclusion by plant roots so that salts do not accumulate in the leaves; and (iii) compartmentalization of salts to avoid toxic concentrations in the cytoplasm, especially the cytoplasm of mesophyll cells in the leaf. Compared with most riparian plants, *Tamarix* has a fairly high tolerance to osmotic stress (Cui *et al*., 2010; Ding *et al*., 2010) and therefore can maintain gas exchange at lower leaf water potentials than most co-occurring species such as willows (*Salix* spp.) or cottonwoods (*Populus* spp.) (Pockman and Sperry, 2000; Hultine and Bush, 2011). More importantly, *Tamarix* avoids long-term salt toxicity in leaf tissues by compartmentalizing and excreting salts through specialized salt glands (Storey and Thompson, 1994; Glenn *et al*., 2012). Compartmentalization takes place by synthesizing compatible organic solutes, such as sucrose and other compounds, at high enough concentrations in the cytosol and organelles of leaf cells to balance the osmotic pressure of ions in the cell vacuole (Flowers *et al*., 1977; Munns and Tester, 2008). However, compatible solute synthesis and subsequent removal of salts once they have entered the leaf come with a considerable metabolic cost resulting in a potential reduction in carbon allocation to other sinks (Fig. 3). To be more precise, about seven moles of ATP are required to accumulate one mole of Na^+^ as an osmoticum, whereas it takes ~52 moles of ATP to synthesize one mole of sucrose (Raven, 1985). The high metabolic cost of solute synthesis may allow plants to survive the presence of high external concentrations of salt, but at the expense of higher vulnerability to other stressors. For example, *Tamarix* dieback and mortality in response to episodic herbivory by *D. carinulata* increased along a soil salinity gradient in a Mojave Desert river watershed (Hultine *et al*., 2015a). Therefore, soil salinity may exert a combination of high osmotic stress (resulting in lower net photosynthesis) and increased metabolic costs (resulting in lower carbon allocation to NSC storage) that may synergistically predispose *Tamarix* to the negative effects of episodic foliage herbivory and other stressors.

Across the geographic range of *Tamarix*, allocation to NSC storage may be selected for in populations that experience freezing temperatures during the growing season. A recent common garden experiment indicated that *Tamarix* genotypes from higher latitudes, where freezing temperatures during the growing season are common, showed greater tolerance to herbivory by *D. carinulata* (i.e. expressed higher canopy regrowth following herbivory) than lower latitude genotypes (Williams *et al*., 2014). One explanation for the observed pattern is that high-latitude genotypes maintained higher NSC storage due to a reduction in sink strength as plant growth ceases at the conclusion of the growing season (Hoch *et al*., 2002). In other words, NSC pools could increase as a consequence of imbalances between carbon supply and demand as growth and respiration decline (Chapin *et al*., 1990; Sala *et al*., 2012). Alternatively, high-latitude genotypes may have been selected to actively maintain high NSC pools. The same common garden experiment revealed evidence for natural selection and subsequent adaptation to local environment conditions as high-latitude genotypes expressed earlier leaf senescence, lower biomass production and higher root-to-shoot ratios compared with genotypes from lower latitudes which exhibited increased aboveground biomass accumulation (Friedman *et al*., 2011; Williams *et al*., 2014). The latter trait is important because the largest NSC pool is presumably stored belowground (Canham *et al*., 1999). Given that high-latitude populations experience occasional canopy dieback associated with freezing temperatures during the growing season, natural selection may favour genotypes that allocate more biomass to belowground NSC storage presumably for rebuilding frost-damaged tissues. As a by-product of selection to cope with low temperatures, high latitude genotypes may be better adapted to tolerate episodic disturbance.

The relative strength of carbon allocation tradeoffs within and among *Tamarix* populations is an open question, but recent evidence suggests that these tradeoffs might be profound. Measurements of radial growth from annual tree rings showed that surviving trees within populations grew slower in years prior to the arrival of *D. carinulata* beetles compared with trees that ultimately succumbed to repeated herbivory events (Hultine *et al*., 2013). These patterns were presumably a function of surviving trees allocating a higher proportion of their carbon pool to NSC storage at the expense of slower annual growth rates (Hultine *et al*., 2013). If plants selected for faster growth are killed by beetles at a higher rate than slower growing plants, then population mean productivity of individuals may decline in response to episodic herbivory. This pattern of directional selection could in turn impact population fitness by altering competitive interactions with other species. This is particularly true given evidence that *Tamarix* already has lower growth rates relative to native riparian tree species *Salix* spp. and *Populus* spp. at germination (Sher and Marshall, 2003).

## Implications for conservation and future research directions

**Figure 4:**
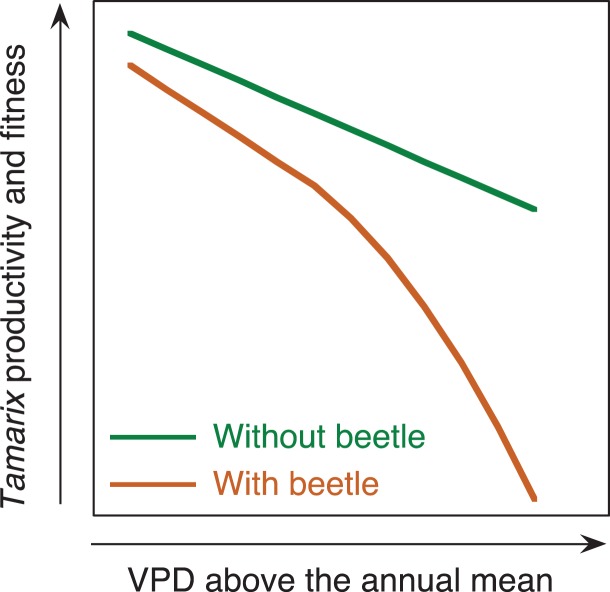
The predicted relationship between growing season aridity (i.e. vapor pressure deficit) above the annual mean and the productivity and fitness of *Tamarix* genotypes with and without the presence of the northern *Tamarix* leaf beetle (*Diorhabda carinulata*).

This review highlights three main points. The first is that despite its recent introduction into North America, *Tamarix* expresses several functional trait variations consistent with adaptation to regional climate conditions, which may be in part a result of extensive hybridization. This result is consistent with previous studies showing local adaptation occurs in invasive plant species (Liao *et al*., 2016, Oduor *et al*. 2016). The second is that *Tamarix* genotypes adapted to warmer climates may be maladapted to the effects of episodic herbivory due to allocating a larger proportion of their total carbon pool to growth and respiration at the expense of lower overall storage of NSCs. Finally, both of these patterns suggest that the combination of herbivory and increased aridity driven by climate change could synergistically reduce growth, fitness and overall dominance of *Tamarix* (Fig. 4). As the distribution of *D. carinulata* and related species continue to expand their range into warmer and more arid regions in North America, rates of *Tamarix* mortality will likely increase compared with current mortality rates in more northern or higher elevation locations. Likewise, *Tamarix* growing under conditions of high soil and/or groundwater salinity may be especially sensitive to episodic herbivory. Plants growing in high salinity will not only be exposed to chronic stress from lower soil water potentials, but will potentially require greater allocation of carbon to solute synthesis in order to osmotically facilitate salt exudation from leaf tissues (Box 1). To better understand how the combination of climate and episodic disturbance (i.e. herbivory) will impact *Tamarix* plants in the southwestern US, we suggest that future research projects take place on four inter-related thrusts: (i) the construction and maintenance of common gardens, such as the one described earlier and currently in operation in Yuma, AZ; (ii) expanded efforts to quantify plant carbon budgets over time, particularly better seasonal accounting of changes in whole-plant NSC pools across genotypes and populations; (iii) genomics and epigenetics research to link phenotypic traits such as resource allocation, tolerance to salinity and drought to specific molecular markers and gene expression, and (iv) ground and remote sensing-based monitoring of *Diorhabda* spp. distributions and subsequent impact on *Tamarix* fitness, recruitment and survival.

Information acquired from current research activities, and research approaches advocated here would provide critical information for targeted restoration and conservation of valued riparian ecosystems. Data on plant genetics and phenotypic trait expression could potentially guide restoration ecologists and land managers on how to best use limited resources for riparian restoration by improving predictive ability based on phenotype/genotype screening on when and where climate change by defoliation interactions will most impact *Tamarix*. These projects could target a wide range of specific objectives depending on the projected impacts of foliage herbivory on *Tamarix* including fire prevention, native plant restoration and habitat restoration. For example, *Tamarix* populations that are predicted to experience the highest levels of mortality and canopy dieback could be areas that are given the highest priority for restoration efforts. Knowing these patterns may be particularly important for guiding habitat restoration of threatened and endangered avian species such as the southwestern willow flycatcher (*Empidomax traillii extimus*) that under some circumstances rely on *Tamarix* canopies for nesting (Bateman *et al*., 2010). It is currently unclear to what extent biological control of *Tamarix* will impact riparian habitat for *E. trallii extimus*. However, the combination of common garden studies of plant resource allocation and stress tolerance with technological advances in molecular genomics will provide critical information on how *Tamarix* populations will likely respond to multiple stressors going into the future.
